# Niemann-Pick Type B: A Rare Cause of Interstitial Lung Disease

**DOI:** 10.7759/cureus.21230

**Published:** 2022-01-14

**Authors:** Rute Sousa Martins, Sara Rocha, Arlindo Guimas, Rosa Ribeiro

**Affiliations:** 1 Internal Medicine, Centro Hospitalar Universitário do Porto, Porto, PRT

**Keywords:** lysosomal storage disorder, respiratory, enzyme replacement therapy, interstitial pulmonary disease, niemann pick type b

## Abstract

Niemann-Pick disease (NPD) is a rare lysosomal storage disease (LSD) with multisystemic involvement. The disease is heterogeneous and classified into three subtypes: type A and B result from deficient acid sphingomyelinase activity and leads to the accumulation of sphingomyelin and type C is a genetically different disease resulting from defective intracellular trafficking of cholesterol with accumulation of glycosphingolipids. Type A is generally a neurodegenerative disease and is fatal in infancy. Type B is a less severe form characterized by pulmonary involvement, hepatosplenomegaly, hyperlipidemia and most patients live into adulthood. In type C, clinical presentation is dominated with neurological involvement. Pulmonary involvement occurs in all three types of Niemann-Pick but most frequently in type B. Clinical manifestations range from a lack of symptoms to respiratory failure, and respiratory symptoms are usually mild with recurrent cough, dyspnoea on exertion and recurrent respiratory infections. Interstitial lung disease (ILD) is the most prominent feature with slow progression, characterized by worsening pulmonary function tests. In recent years, enzyme replacement therapy has shown promising results in clinical trials, such as improvement in organomegaly and pulmonary involvement with the potential to improve patients’ lives. We present three cases of NPD with pulmonary involvement, each exhibiting a different pattern of ILD and evaluate therapeutic options.

## Introduction

Niemann-Pick disease (NPD) is a rare lysosomal storage disorder (LSD) inherited in an autosomal recessive pattern. Lysosomes are cellular organelles involved in degrading and recycling cellular waste, cellular signalling and energy metabolism. Lysosomal diseases are the result of defects in genes encoding lysosomal proteins which in turn makes it impossible to degrade molecules like glycolipids, glycoproteins, mucopolysaccharides or to release the products of their catabolism in cytosol [1].

NPD is classified into three subtypes: type A, B and C. NPD types A and B affect 1 in 250,000 individuals with the prevalence being higher in Ashkenazi Jewish descent, where it affects 1 in 40,000 individuals, and NPD type C affects 1 in 150,000 people [2]. Types A and B result from deficient acid sphingomyelinase (ASM) activity due to mutations in sphingomyelin phosphodiesterase 1 (SMPD1) gene which leads to accumulation of sphingomyelin (a major component of cell membranes and a principal phospholipid of the myelin sheath), primarily in tissues of the reticuloendothelial system [2-4]. NPD type C is a genetically distinct disease caused by mutations in two distinct cholesterol-binding proteins (NPC1 and NPC2) leading to impairment movement of lipids out of the cells and to their secondary accumulation within the cells [3].

Symptoms are caused by the accumulation of lipid-laden macrophages in various organs such as liver, spleen, lung, lymph nodes, adrenal cortex, central nervous system and bone marrow. NPD type A presents in the first few months of life, usually in the first six months of age, as hepatosplenomegaly and growth retardation [2]. Neurological symptoms appear as psychomotor retardation and even rapid regression of developmental milestones. These children usually do not survive past three years often due to respiratory failure [4,5]. NPD type C can present at any age but usually during childhood and affected individuals can have visceral, neurological and psychiatric manifestations mainly: ataxia, dystonia, supranuclear gaze palsy, dysphagia, progressive cognitive decline and severe liver and lung disease [2,6].

NPD type B usually presents in mid-childhood and is not as severe as type A. Clinical presentation and disease course are variable, making it possible for the disease to be diagnosed late as the sixth decade of life [4]. The most common presentations are hepatosplenomegaly, interstitial lung disease (ILD) causing recurrent lung infections, haematological manifestations with thrombocytopenia and leukopenia, slowed bone growth and even decreased bone mineral density [3]. Typical NPD type B do not exhibit neurological manifestations but a subset of these patients may have variable degrees of peripheral neurological symptoms [5].

Respiratory involvement occurs in all three types of NPD but most frequently in type B and tends to be the major cause of mortality and morbidity in these patients [3,4,7]. Respiratory symptoms are highly variable ranging from, asymptomatic to non-specific and respiratory failure.

Respiratory symptoms, lung parenchyma changes and alterations of pulmonary function tests are not specific to NPD. Other lysosomal storage diseases should be kept in the differentials, especially Gaucher disease type III which can also present as ILD, hepatosplenomegaly and cytopenias, although bone pain and lesions are more prominent. A deficiency of acid glucocerebrosidase resulting in abnormal accumulation of glycolipids within lysosomes are responsible for this disease.

In the presence of clinical features suggestive of NPD, diagnosis of NPD types A and B can be made with the demonstration of reduced ASM activity in leukocytes or cultured skin fibroblasts, or with genetic testing with mutations on SMPD1 gene. In type C, measurement of oxysterols is the first-line screening test and diagnosis is confirmed with genetic testing showing mutations in NPC1 or NPC2 genes [8].

Enzyme replacement therapy (ERT) is one of the main treatments for enzyme delivery to organs affected by LSD. As in other LSD, this type of therapy has no effect on the progression of neurological disease; recently Olipudase alfa (a recombinant human acid sphingomyelinase) used in NPD type B showed improvements in ILD, spleen and liver volumes and quality of life [9].

We present three case reports illustrating respiratory impairment and the importance of this differential diagnosis in the presence of ILD since the advent of an available treatment with improvement in prognosis.

## Case presentation

Case report 1

**Figure 1:**
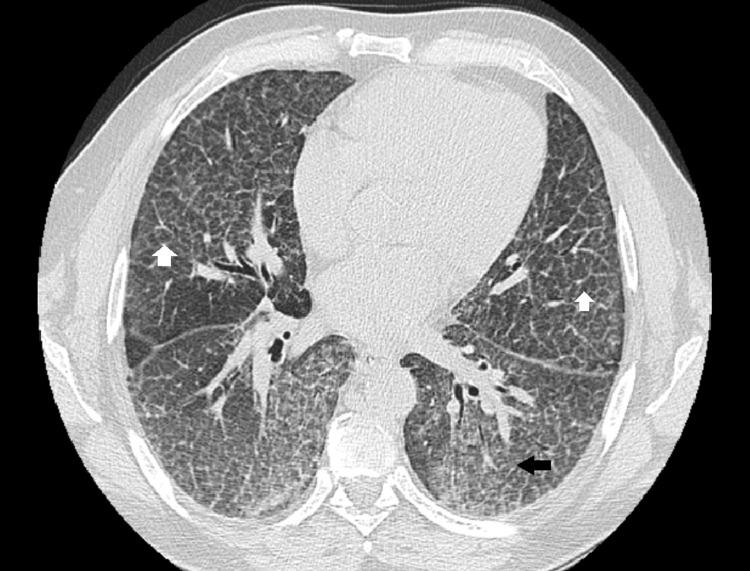
Thoracic CT scan showing ground-glass opacities (black arrow) with interlobular septal thickening (white arrows).

A 59-year-old man was referred to our centre with dyspnoea for moderate to light exercise. He had this symptom for nine years with no specific diagnosis and anaemia of unknown cause for three years. During follow-up for anaemia, he had spontaneous spleen rupture with histology compatible with NPD; ASM activity measured in fibroblast was low and genetic test with two mutations confirmed the diagnosis of Niemann-Pick type B. On admission, he had shortness of breath for moderate exercise but not at rest. Blood arterial gases reveal a ratio pO_2_/FiO_2_ below 300. He had never smoked. The thoracic computed tomography (CT) scan found ground-glass opacities with interlobular septal thickening (Figure 1), characteristic of the so-called “crazy-paving” pattern.

Pulmonary function test revealed restriction (Forced Vital Capacity 87% of predicted, Total Lung Capacity and Residual Volume below the lower limit of normal, with normal FEV1/FVC ratio) with lowered diffusion capacity for carbon monoxide (DLCO) (58% of predicted). 6-minutes’ walk distance test performed under continuous 4 L/min oxygen showed decreased saturation down to 84% with 348 m distance walked (58% of predicted value for a healthy patient). Abdominal CT scan showed hepatomegaly with normal liver contour and heterogeneous liver parenchyma, suggestive of steatosis with otherwise unremarkable findings in the remaining organs. To evaluate the cardiovascular involvement, echocardiography was performed: no signs of diastolic or systolic dysfunctions with preserve ejection fraction and no indirect sign of pulmonary hypertension. Liver function was normal and no signs of portal hypertension was found.

The possibility of lung transplant was discussed with the patient, since he has advanced and progressive lung disease for which medical therapy is unavailable, but he refused this type of treatment because he is afraid of an immunosuppression state. Then, the medical team proposed this patient for ERT and he is waiting for the availability for Olipudase alfa treatment.

Given the prior diagnosis of NPD, a bronchoscopy (with bronchoalveolar lavage) was not performed on the patient, nor a lung biopsy, but a thorough patient medical history and physical examination were done, with no additional findings suggestive of other diseases. Autoimmune diseases, tuberculosis and sarcoidosis were also excluded with additional laboratory work. 

In the follow-up, thoracic CT scan and pulmonary function tests show slow deterioration of lung function. He remains active and is still able to work.

Case report 2

**Figure 2:**
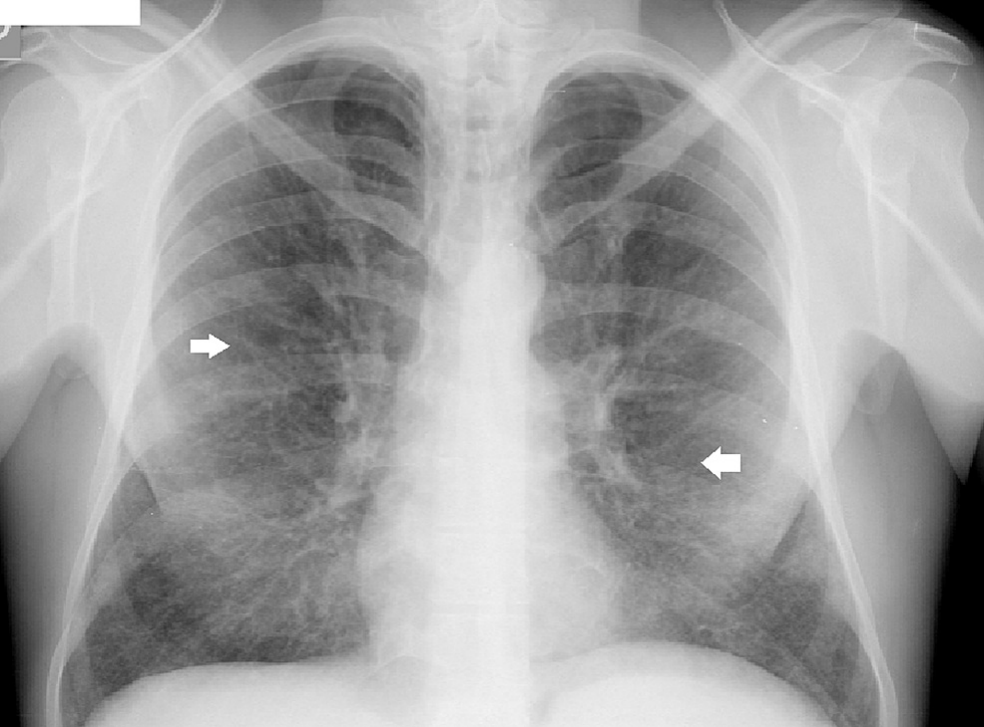
Chest radiography shows reticular infiltrates (white arrows).

**Figure 3:**
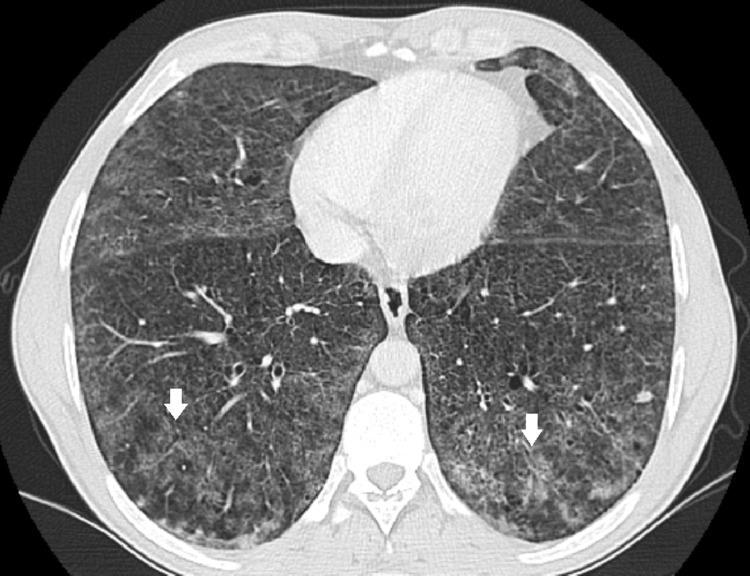
CT scan showing bilateral lower lobes ground glass opacities associated with intermixed interlobular lines in lower lobes (white arrows).

**Figure 4:**
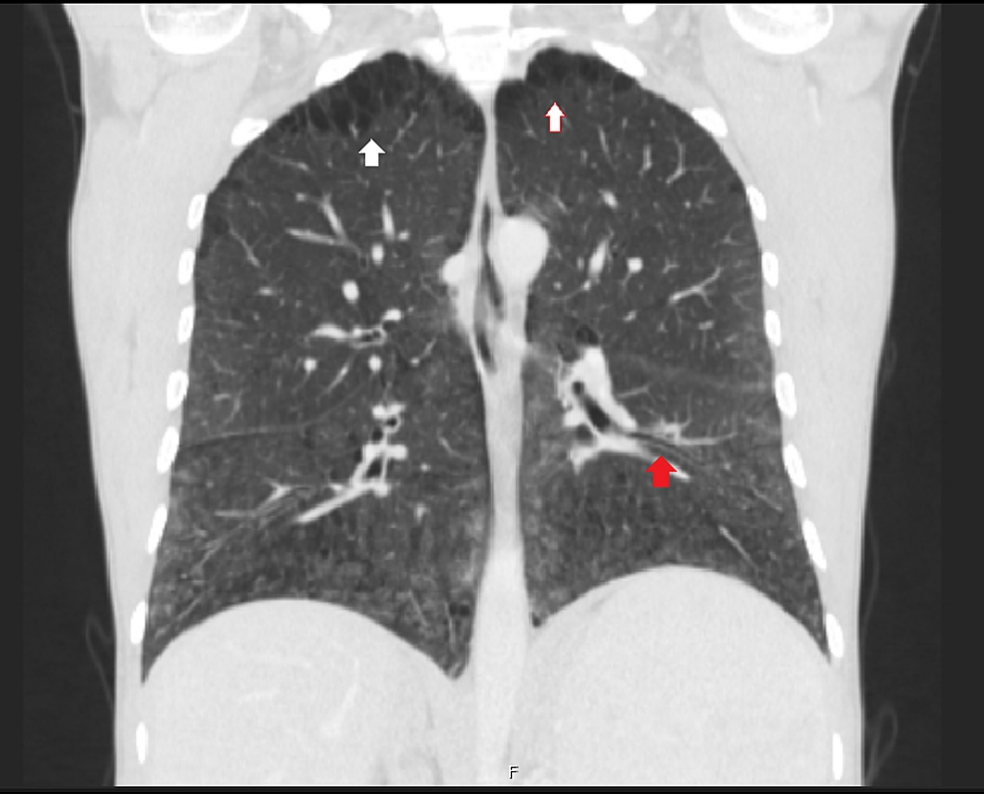
Emphysema lesions in upper lobes (white arrow) and lower lobes with bronchiectasis (red arrow).

**Figure 5:**
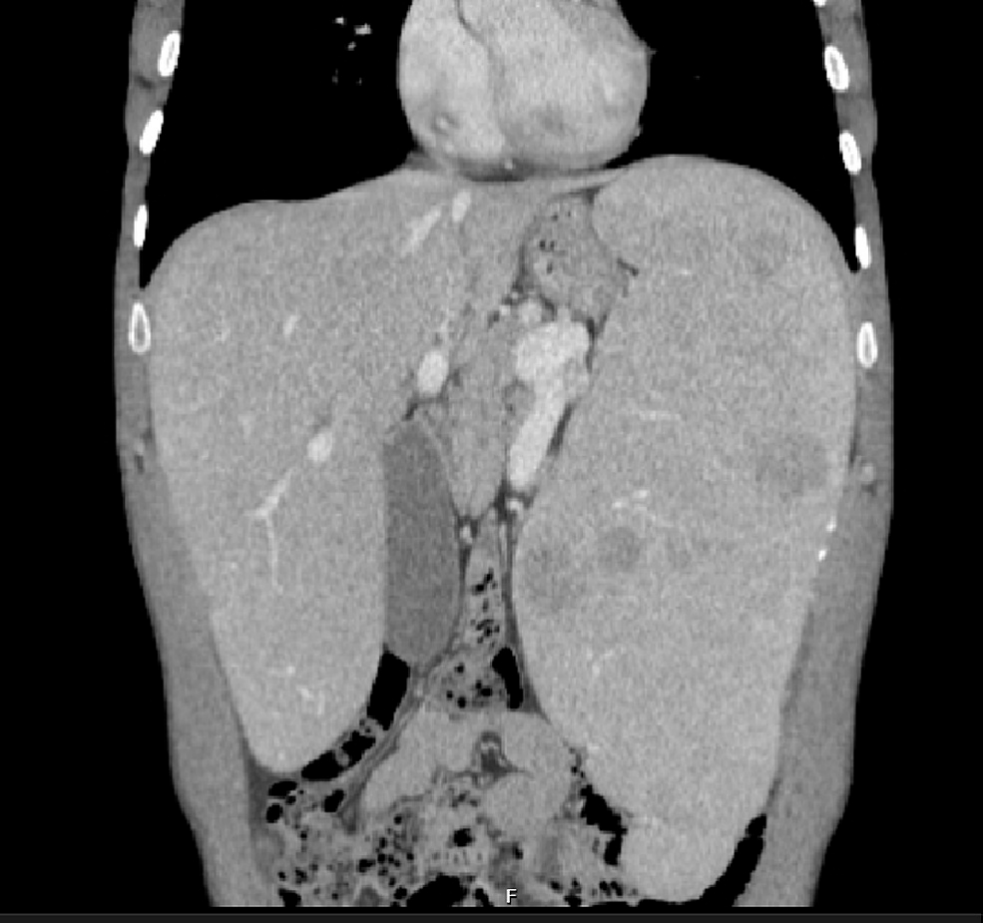
CT scan showing hepatosplenomegaly.

A 43-year-old man diagnosed with Niemann-Pick type B was referred to our centre for follow-up. He was diagnosed at 13 years old following the study of hepatomegaly and impaired growth. He had a history of multiple respiratory infections as a child and had a history of smoking (30 pack-years). He has no respiratory symptoms and does daily training in a gymnasium. Laboratory findings show low platelets count (92.000 μL), normal haemoglobin levels, normal renal and hepatic function. Cholesterol and triglycerides levels are within the normal range with no medication. Chest radiography shows reticular infiltrates (Figure 2) and thoracic CT scan showed bilateral lower lobes ground glass opacities associated with intermixed interlobular lines in upper and lower lobes (Figure 3). Centrilobular and paraseptal emphysema lesions were observed predominantly in the upper lobes whereas discrete distal bronchiectasis was noticeable in the basal lungs (Figure 4). Hepatomegaly and splenomegaly were also found (Figure 5).

Pulmonary function test showed an obstructive pattern (Tiffeneau-Pinelli index 68%) with lowered diffusion capacity for carbon monoxide (41% of predicted). No alterations were found in echocardiogram. Before the follow-up in our centre, he has performed a bronchoscopy with bronchoalveolar lavage that, according to the medical reports, showed an increase of the total number of cells and the presence of ‘‘foamy’’ macrophages called Niemann-Pick cells. Although this finding isn’t pathognomonic of the NPD, as it can be found in other lysosomal storage diseases, given the previous diagnosis, it was presumed that the pulmonary alterations were related to NPD. No lung biopsy was performed but tuberculosis, as well as autoimmune diseases, were excluded.

The patient shows a slow degradation of pulmonary function, but without hypoxemia or respiratory symptoms over the following years. Since the patient is asymptomatic, it was decided to maintain active surveillance and periodic reassessment of lung function. The patient maintains daily activity in the gym without reporting any restrictions.

Case report 3

**Figure 6:**
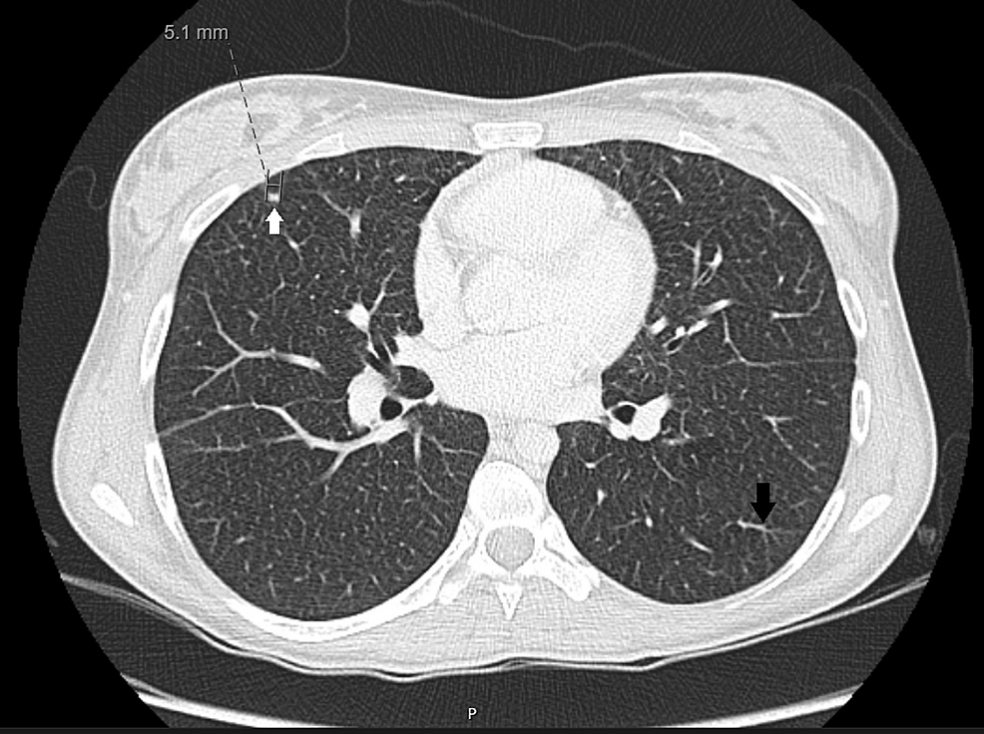
Discrete interlobular septal thickening (black arrow) and subpleural calcified micronodules (white arrow).

A 30-year-old woman is followed for nine years in our centre with the diagnosis of Niemann-Pick type B, after being followed in paediatrics centre since two years old. As a child, she had impairment growth with short stature and splenomegaly. She was diagnosed with Niemann-Pick type B based on clinical and molecular diagnosis with low level of ASM. As an adult, she doesn’t have any respiratory or neurological symptoms. Laboratory findings of hypertriglyceridemia and high LDL-cholesterol and thrombocytopenia were found. Densitometry shows osteopenia. Thoracic CT scans have discrete interlobular septal thickening and calcified micronodules (Figure 6).

6-minutes’ walk distance test performed with no supplemental oxygen, showed non-significant desaturation after almost 500-m distance walked and pulmonary function test has normal lung volumes and a small decrease in DLCO (73% of predicted). She remains stable and wants to get pregnant. The genetic diagnosis was made at 26 years old with two mutations found in SMPD1 gene. Genetic testing was also given to her partner in order to assess the risk of transmission of the disease.

The patient still has no respiratory symptoms and pulmonary alterations remain stable as do pulmonary function.

## Discussion

Niemann-Pick type B is a heterogenous disease with multiple possible presentations. Our three cases have pulmonary involvement with different degrees of involvement and respiratory symptoms. Usually when present, respiratory symptoms are mild with recurrent cough, exertional dyspnoea and recurrent infections but rapidly fatal and progressive lung disease have also been reported [1]. Progression of lung disease is slow but unavoidable, due to the accumulation of ASM in lung cells. It is also important to refer that at some extent the respiratory symptoms can be aggravated or even caused by hepatosplenomegaly.

According to a study with 53 patients [10] with NPD type B, 98% of patients exhibit ILD on CT scan. The most common findings are ground-glass opacities (predominantly in upper lung zones), interlobular septal thickening (predominantly in lower lung zones) and intralobular lines, like in our patients. Although rare it is possible to find cysts, nodular opacities (with calcifications), peribronchovascular thickening, segmental atelectasis, bronchiectasis and emphysema [10,11] as in case report 3.

In the presence of ILD of unknown cause, bronchoscopy with bronchoalveolar lavage can be very helpful as it confirms the lung involvement and, in the presence of other findings suggestive of NPD (such as enzyme testing, gene sequencing and biomarkers), can avoid a lung biopsy [1,3,7]. In this disease, the bronchoalveolar lavage typically shows increased cell numbers and large multivacuolated histiocytes containing granules stained deep blue with May-Grunwald-Giemsa stain (Niemann-Pick cells) [1,5]. Although important for the diagnosis these cells are not pathognomonic and can appear in other lysosomal storage disease like Gaucher disease. A lung biopsy when performed, shows numerous vacuolated foamy macrophages located within the alveoli and to a lesser extent, thickening of the alveolar walls [11]. Vacuolation of bronchial ciliated epithelium, without vacuolation of type 2 pneumocytes and mild inflammation with interstitial fibrosis may also be observed [11].

Pulmonary function tests are useful to evaluate the consequences and to assess the progression of accumulation of sphingomyelin in the lung cells. There is a poor correlation between the extent of radiological abnormalities and pulmonary function test changes [10] implying that interstitial changes do not necessarily mean affected gas exchange (as in our case 2). Like in other interstitial lung diseases, the most characteristic finding is the decrease of the lung compliance and DLCO. Longitudinal studies show slow but progressive deterioration of pulmonary function tests, reflecting the progression of the disease [12,13] as was seen in our case reports.

Hepatosplenomegaly is a prominent finding in patients with NPD and although rare, hepatic involvement can lead to cirrhosis and hepatic failure [13]. In many patients this finding along with abnormalities in haematological lineages are the triggers for an etiologic investigation but, due to the rarity of this disease, the diagnosis can be missed. In our case report 1, the patient had had dyspnoea and anaemia for a long time but the diagnosis was made only after spleen histology.

It is also important to remind the skeletal involvement in NPD type B not only in concerns of growth abnormalities (mainly due to abnormalities in the IGF-1 signalling pathway [4]) but also because decreased bone mineral density makes patients more vulnerable to have fractures (including rib fractures) [14] that can add more morbidity in terms of pulmonary function.

No cure exists for NPD. Supportive care is the mainstay of treatment. For patients with pulmonary involvement, oxygen and physical therapy can be provided. Organ transplant (bone marrow and lung transplant) has also been done but only in a few cases and with low rate of success [4,5]. Whole lung lavage was efficient in a few cases but is a high-risk procedure with short-term benefits [7]. Whole-lung lavage was used in NPD patients with aggravated respiratory function and the rationale behind this treatment is based on the precedent of successful treatment of other alveolar filling disorders, such as alveolar proteinosis [15]. According to reports, whole-lung lavage results in significant symptomatic and radiographic improvements by reducing the surfactant impairment and the alveolar filling due to the presence of foamy cells [16].

Given the pathophysiology behind this disease, enzyme replacement therapies may become the mainstay of treatment in the future. Olipudase alfa, a recombinant human acid sphingomyelinase, is an enzyme replacement therapy for the treatment of non-neurologic manifestations of acid sphingomyelinase deficiency that has been showing promising results [4,15]. The ASCEND trial [17], a 52-weeks, multicenter, randomized, double-blind, placebo-controlled trial evaluated the efficacy and safety of Olipudase alfa in 36 adults with chronic visceral NPD. The primary endpoints were the percent change in spleen volume and percent-predicted diffusing capacity of the lung for carbon monoxide. At week 52, treatment with Olipudase alfa resulted in a 39.5% reduction in spleen volume, compared with a 0.5% increase for placebo (p < 0.0001). A decrease in spleen volume of at least 30% was observed in 17 patients (94%) treated with Olipudase alfa compared with no patients treated with placebo. Additionally, this therapy, significantly improved lung function by 22% from baseline compared with 3% for the patients receiving placebo (p = 0.0004), as measured by percent predicted DLCO. Olipudase alfa also met key secondary endpoints including a 31.7% reduction in liver volume (vs a 1.4% reduction for placebo; p < 0.0001) and a 16.8% improvement in mean platelet counts (vs 2.5% with placebo; p = 0.019) at week 52 [17].

Long-term effects of Olipudase alfa are still unknown [9], but the major improvement in the primary end-points after one year of treatment, suggests a major breakthrough for these patients. Olipudase alfa is not currently available but compassionate use is possible. Since the major causes of death in NPD type B disease are respiratory failure and liver failure [14,18] early treatment with ERT can prevent mortality and improve quality of life.

## Conclusions

Niemann-Pick disease is a rare systemic disease where pulmonary manifestations like interstitial lung disease can be common. Early diagnosis is still lacking, mainly because of unawareness of this disease from the healthcare professionals. There aren’t any pathognomonic symptoms or signs and in the presence of hepatosplenomegaly combined with interstitial lung disease a storage disorder must be thought.

The cases discussed in this review show the need to treat the patient as a whole and incite professionals to be aware that rare inborn diseases can also present in adult patients and early detection can in some cases prevent bad outcomes. Management of Niemann-Pick disease type B is currently based on symptomatic and supportive care and the disease is with no doubt progressive, but recent ERT results can bring a new hope for improving prognosis, quality of life and extend life expectancy. A multidisciplinary team should be on board as soon as the diagnosis is made to improve the probability of success.
